# Study on the Optimization of *b*-Value for Analyzing Weld Defects in the Primary System

**DOI:** 10.3390/s24237456

**Published:** 2024-11-22

**Authors:** Do-Yun Jung, Young-Chul Choi, Byun-Young Chung

**Affiliations:** Korea Atomic Energy Research Institute, Daejeon 34057, Republic of Korea; cyc@kaeri.re.kr (Y.-C.C.); cby@kaeri.re.kr (B.-Y.C.)

**Keywords:** ALMS, acoustic emission testing, felicity effect, *b*-value, RMS

## Abstract

This study presents a method to add a crack analysis algorithm to the Acoustic Leak Monitoring System (ALMS) to detect and evaluate the crack growth process in the primary system piping of nuclear power plants. To achieve this, a fracture test was conducted by applying stepwise loading to welded specimens that simulate the cold leg section, and acoustic emission (AE) signals were measured in relation to the increase in strain using an AE testing system. The experimental results indicated that the stability and instability of cracks could be assessed through the Kaiser effect and the Felicity effect when detecting crack growth using AE signals. Additionally, by utilizing both root mean square (RMS) and amplitude parameters simultaneously to calculate the *b*-value, it was confirmed that the RMS-based *b*-value minimizes the effects of AE signal attenuation and allows for a more stable assessment of crack progression. This demonstrates that the RMS, which reflects signal energy, is effective for real-time monitoring of the crack growth state. Finally, the results of this study suggest the potential for real-time crack monitoring using AE data in piping systems of critical structures, such as nuclear power plants; by adding a simple AE analysis method to the ALMS system, a practical approach has been derived that enhances the safety of the structure and allows for quantitative assessment of crack progression. Future research is expected to further refine the AE parameters and algorithms, leading to the advancement of safety monitoring systems in various industrial settings.

## 1. Introduction

Pipelines must meet strict safety standards and maintain consistent quality. In particular, rigorous quality control is essential for high-risk piping systems, such as the primary system in nuclear power plants. However, long-distance monitoring faces challenges due to the complexity of maintenance. To address this issue, a reliable condition-monitoring method capable of detecting anomalies, such as leaks, has been studied, leading to the development of Acoustic Leak Monitoring Systems (ALMS) [1]. This system is based on the Leak-Before-Break (LBB) concept, introduced in the 1970s in the nuclear industry to ensure the safety of piping systems. It plays a crucial role in enhancing pipeline reliability by detecting cracks before they lead to failure, thereby preventing accidents [2,3].

In fact, an LBB event on 27 March 2017, involving a coolant leak of approximately 413.86 L from the drain valve on the ‘A’ side of the steam generator in Kori Nuclear Power Plant Unit 4, supports this. The investigation revealed a 6 mm deep and up to 10 mm long through-wall crack in the socket weld. The cause of the crack was identified as vibration fatigue accumulation due to changes in the natural frequency of the drain pipe resulting from design modifications, leading to vibration and resonance. Consequently, both short-term and long-term measures were implemented to prevent recurrence, and the plant has since been operating without technical issues. This case highlights the need for analysis methods that enable early detection and diagnosis of cracks occurring before leakage.

The locations of Acoustic Emission (AE) sensors are determined through computational structural analysis; typically, about 20 sensors are installed near welds and valves. These locations are crucial for detecting early defects because system anomalies are more likely to originate near welds and valves [4]. Signals emitted from cracks and leaks propagate along the surface of the pipe, attenuating as they travel. According to a study by Graham and Alers, in pressure vessel testing, the amplitude decreases linearly with propagation distance, but the rate of decrease is faster near the signal source [5,6]. This is because the signal energy is consumed intensively at close range. ALMS with AE sensors installed near the welds (and valves) can effectively detect the presence of cracks or leaks, but they have limitations when it comes to quantitatively evaluating these defects. For example, it is difficult to predict the optimal maintenance timing because the size and growth rate of cracks cannot be identified. Similarly, the inability to quantify the amount and rate of leakage makes it challenging to accurately assess the severity of leakage risks.

AE is defined by ASTM E1316 (2022) as a “transient elastic wave generated by the rapid release of energy from a localized source within a material or a transient wave generated thereby [7]”. AE is a non-destructive testing (NDT) method that collects and analyzes signals generated during deformation or fracture in real time to detect structural defects. We conducted a review of studies where AE technology has been applied to diagnose cracks in metallic materials. Chai Mengyu [8] evaluated fatigue crack growth using AE entropy in a 2018 study on 316LN stainless steel. In 2022, Chai conducted a study that identified and predicted fatigue crack growth under various stress ratios based on AE data, demonstrating the potential of AE technology for fatigue crack monitoring [9,10]. Zhifen [11] analyzed the effects of mixed loading mode (I + II) on the mechanical properties and AE parameters of 30CrMnSiA structural steel, demonstrating that the change patterns of average frequency and rise angle can distinguish between brittle and ductile fracture mechanisms. Sulochana Shrestha studied a method for distinguishing cracks through frequency analysis of AE signals and suggested that high peak frequencies (above 400 kHz) indicate major planar cracks, while low peak frequencies (below 200 kHz) represent micro-cracks. Lu Yang [12] monitored fatigue damage in duplex 2205 stainless steel under various stress ratios using AE technology and demonstrated that AE parameters, such as count and amplitude, can effectively characterize the three stages of fatigue crack development: initiation, stable growth, and failure. Lee [13] analyzed AE signals during tensile tests on magnesium alloy (AZ31B) sheets and used the Gaussian Mixture Model (GMM) algorithm to distinguish between yielding and fracture. He observed that high-energy signals with greater amplitude and energy occur within a short period during fracture. These studies suggest that AE technology is a valuable tool for understanding the progression of fatigue cracks and the nature of structural defects [14,15,16]. A single AE sensor is installed in the primary system, focusing on welds and valves. By analyzing the AE signals measured through this setup, it is possible to identify the progression of fatigue cracks, the type of failure, and the current stage of development. These studies suggest that AE technology is a valuable tool for understanding the progression of fatigue cracks and the nature of structural defects. In particular, by installing a single AE sensor on the welds and valves of key systems, the analysis of measured AE signals can identify the progression of fatigue cracks, failure types, and current development stages.

It also estimates the source location by analyzing the time differences of signals arriving at multiple sensors [17]. According to Wadley et al. [18], AE provides valuable insights into understanding the dynamic behavior that occurs during deformation and fracture processes. This enables precise identification of defect locations, contributing to structural stability assessment. In structural health monitoring (SHM) using AE, indicators such as signal amplitude, AE hits, and root mean square (RMS) are used. These indicators reflect the intensity, frequency, and energy of AE events, making them useful for evaluating AE activity levels [19]. These indicators play a crucial role in detecting and monitoring structural damage or defects, such as reinforcement steel corrosion [20], steel pipes [21], and pipeline leakage [22], by using multiple AE sensors to locate the source and analyzing AE parameters. However, since only a single sensor is used in the primary system, parameter selection or correction through location estimation is not feasible. Therefore, a new analytical method is required for ALMS.

A similar issue also occurred in seismology, and Gutenberg addressed it by using the *b*-value [23]. The *b*-value represents the relationship between the frequency and magnitude of earthquakes, making it useful for understanding seismic activity. It can also be applied to AE events. By statistically analyzing the frequency and magnitude of AE events, the severity of cracks or leaks can be quantitatively evaluated. The reason the *b*-value is unaffected by location is that it is based on the statistical distribution of events with varying magnitudes occurring within a specific region. To explain in more detail, this method involves using amplitude, which attenuates similarly with propagation distance, and AE hits, which represent the number of AE signals exceeding a certain amplitude, to create a frequency distribution. The slope of this distribution (a dimensionless constant) is then used to eliminate the effect of the attenuation rate. Therefore, in ALMS, it is possible to analyze AE signals using statistical methods like the *b*-value, enabling consistent evaluation of cracks and leaks regardless of location. According to Antorino’s study [24], the traditional *b*-value calculated based on amplitude was successfully replaced with AE energy and applied to reinforced concrete structures. The evolution of the energy *b*-value indicates the progression of severe damage in critical areas of the structure (e.g., beam-column joints) during loading processes, demonstrating its promising use in structural health monitoring. Therefore, in ALMS, which evaluates leaks and cracks based on RMS, it is necessary to develop a new analytical approach by referencing this method.

**Figure 1:**
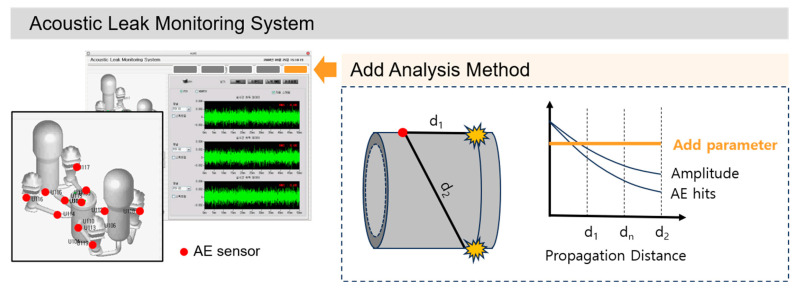
Overview of the ALMS system and research objectives.

The primary objective of this study is to develop a new crack analysis algorithm based on AE testing and the ALMS system to detect and assess the crack growth process in the primary system piping of nuclear power plants (Figure 1). To achieve this, we first evaluated the feasibility of monitoring cracks using the *b*-value and improved the analysis method by utilizing the AE parameter (RMS) recorded in the ALMS. This approach adds a simple analysis method to the ALMS, providing a new function to assess crack stability, thereby contributing to maintaining the safety of the related structures.

## 2. Experimental Details

Leakage events in the primary system of nuclear power plants primarily occur at welds or valves. In this study, fracture tests and AE tests were designed to replicate the welds. Through this, we aim to verify the applicability of the *b*-value and optimize the *b*-value for the primary system. This study was conducted using welded metal specimens simulating drain valve welds, with the main objective of evaluating the applicability of the crack assessment method using the *b*-value. To clearly achieve this goal, fracture tests were performed, excluding the operating conditions of the primary system, and the optimization of the *b*-value considering environmental factors (noise, temperature fluctuations) will be discussed in the next phase. The detailed content related to this study is explained in the following chapters.

### 2.1. Material and Specimens

SM45C was chosen to simulate the welded joint of the valve in the primary system made of STS. This is because both materials have similar densities (SM45C: 7850 kg/m^3^, STS 316: 7990 kg/m^3^) and elastic moduli (SM45C: 200 GPa, STS 316: 193 GPa), which are expected to result in comparable AE signal propagation and attenuation characteristics. Additionally, SM45C has excellent mechanical properties and machinability, making it suitable for replicating experimental conditions, thereby reliably reproducing the AE signal generation mechanisms that could occur in STS316 piping.

**Figure 2:**
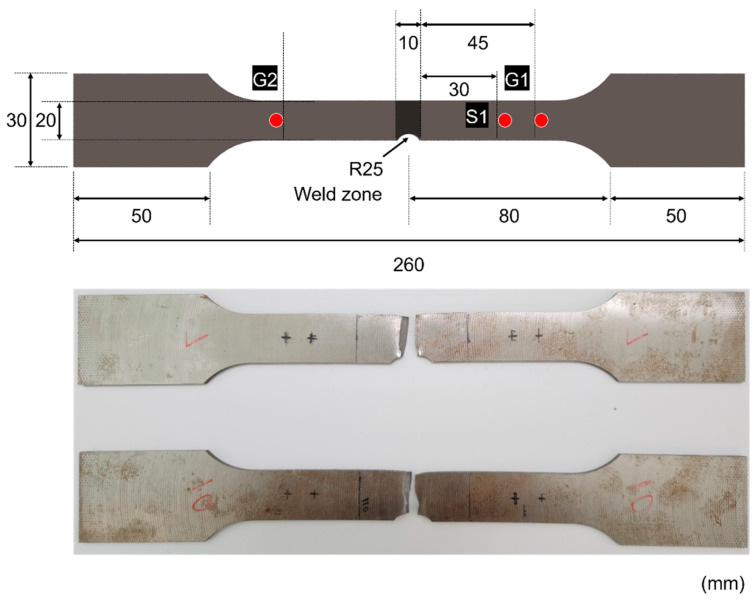
Shape and dimensions of the weld joint: The red circles in the figure indicate AE sensors. S1 is the sensor used for measurement, while G1 and G2 are guard sensors installed to eliminate noise.

In this study, SM45C structural steel with a thickness of 5 mm, produced by POSCO (Pohang-si, Republic of Korea), was used. The specimen was cut into a rectangular plate and then butt-welded. The welding rod used was TGC-50 (Chosun welding Co., Ltd., Pohang-si, Republic of Korea), and welding was performed using an argon welding process with argon gas as the shielding gas. The surface of the specimen was milled to a thickness of 3 mm, and the surface roughness (R_max_) was adjusted to a range between 12.5 and 25S. Subsequently, the specimen was fabricated into a dog-bone shape through laser machining; the detailed dimensions are presented in Figure 2. The specimen conforms to ASTM E8/E8M, the standard test method for tensile testing of metallic materials [25].

### 2.2. Acoustic Emission Monitoring

**Figure 3:**
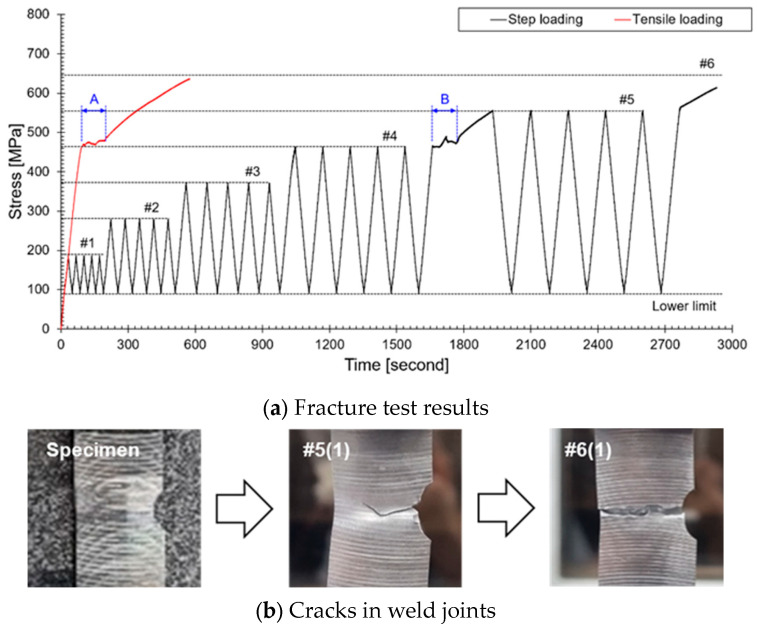
Fracture test results: (**a**) step-loading design dividing the maximum tensile load into six stages, (**b**) image showing crack growth progression at each stage, with the monitoring target up to the flow region (A,B).

The welded specimens were tested under tensile load and performed in displacement control mode (1 mm/min), as recommended by the ASTM E8 standard [25]. The deformation induced by the load was recorded through a uniaxial strain gauge attached to the effective gauge length on the surface of the specimen. The black line in Figure 3 represents the results of the tensile test conducted using the INSTRON 5985 (INSTRON, Norwood, MA, USA) universal testing machine. In the tensile test, the yield strength was measured at 462 MPa, and the tensile strength at 640 MPa. The tensile strength was divided into six stages (minimum: 100 MPa, maximum: #1: 190 MPa, #2: 280 MPa, #3: 370 MPa, #4: 460 MPa, #5: 640 MPa) for step-loading tests. During step-loading, the yield strength was 460 MPa, and shear occurred at the first load of the sixth stage (614 MPa). Each step was repeated five times for fracture testing, and AE (Acoustic Emission) testing was conducted during the test process. AE signals were simultaneously recorded using the PAC PCI-2 data acquisition system. A piezoelectric sensor with a frequency range between 50 kHz to 750 kHz was mounted at the center of the specimen; detailed information on the AE sensor and preamplifier is presented in Table 1. To improve AE signal acquisition, a thin layer of silicone gel and vinyl tape was applied between the sensor and the surface of the specimen. The silicone gel contributed to preventing the recording of reflected signals. The threshold frequency for AE signal acquisition was set at 40 dB_AE_, and signals exceeding this threshold were amplified to 40 dB_AE_ through 2/4/6 AE preamplifiers (MISTRAS, Princeton Junction, NJ, USA). The waveform was recorded at a sampling rate of 10 MSPS.

### 2.3. b-Value Analysis

The *b*-value analysis is a method for quantitatively evaluating damage progression through the amplitude–frequency distribution of AE (Acoustic Emission) signals. This analysis is based on the Gutenberg–Richter law and examines the relationship between the frequency and amplitude of AE events on a logarithmic scale [24]. The *b*-value is calculated using the following equation:

(1) $$\log_{10}\left(N\right)=a-b\times dB_{AE}$$

Here, N is the number of AE events occurring within a specific amplitude range, $dB_{AE}$ is the peak amplitude of the signal in decibels, and a and b are empirical constants. $dB_{AE}$ is converted from the maximum amplitude of the AE signal ($A_{max}\;)$) as follows:

(2) $$dB_{AE}=20\times \log_{10}A_{max}\;$$

**Figure 4:**
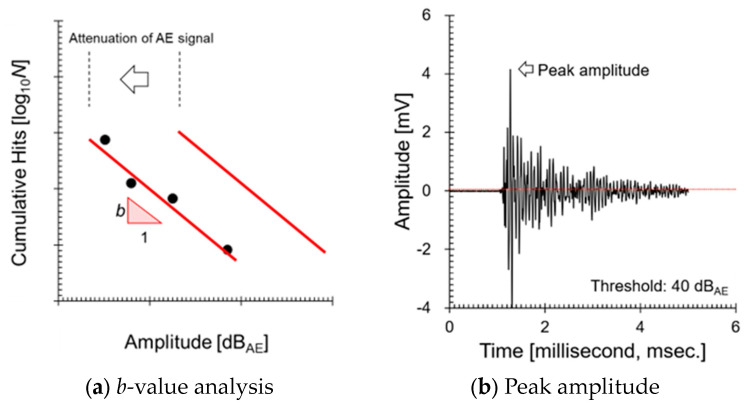
Description of *b*-value analysis: (**a**) *b*-value analysis method, (**b**) AE signal amplitude used in the analysis to evaluate crack progression.

To calculate the *b*-value, AE data are first collected, and the events are grouped into a fixed number (e.g., 100 events, or 10 events in this study). Then, as shown in Figure 4, a log amplitude–frequency graph is plotted for each group, and linear regression is performed using the least-squares method to obtain the slope. This slope is the *b*-value. The *b*-value tends to be higher when there are many micro-cracks and lower when macro-cracks occur. Salvatore Salamone [26] progressively collapsed Reinforced Concrete (RC) shear walls, collected AE signals, and calculated the *b*-value. The *b*-value decreased when macro-cracks occurred and increased when micro-cracks formed. This phenomenon is also observed in studies on the *b*-value or modified *b*-value. As mentioned earlier, AE parameters change according to the size of the crack. Specifically, when micro-cracking is dominant, many small cracks form at the crack tip, resulting in the emission of numerous low-amplitude signals. This phenomenon results in signal accumulation in the low-amplitude range of Figure 4a, during which the *b*-value increases. Conversely, when macro-cracking is dominant, large signals that penetrate micro-cracking are emitted in smaller numbers, leading to a decrease in the *b*-value. These two changes can be used to determine whether a crack is growing. Additionally, since the *b*-value uses the maximum AE signal measured by the sensor and the number of AE signals exceeding a certain amplitude, as shown in Figure 4b, the distribution of AE amplitudes shifts to the left due to propagation distance, as shown in Figure 4a, but the slope represented by the *b*-value is not affected. In metallic materials, *b*-value analysis is used to monitor fatigue damage and crack propagation. In aircraft components, AE events are detected during fatigue testing to track the progression of cracks and prevent unexpected failures [27]. This technique is used not only in Structural Health Monitoring (SHM) [28] but also in rock mechanics [29] and seismic monitoring [30]. In addition, the *b*-value serves as an essential tool for early detection of damage progression in post-earthquake aftershock analysis [31], landslide prediction [32], and the safety assessment of mines and tunnels [33]. This enables both safety assurance and efficient maintenance in high-risk environments.

## 3. Results and Discussion

**Figure 5:**
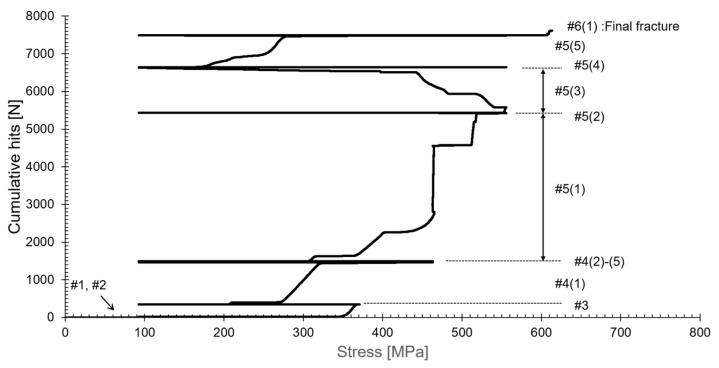
Representation of the Kaiser effect and Felicity effect using AE signals obtained from step loading tests.

The objective of this study was to integrate a crack analysis algorithm into the ALMS system to detect and evaluate the crack growth process in the primary system piping of nuclear power plants. To achieve this, a fracture test was conducted by applying stepwise loading using welded specimens that simulate the primary system piping of nuclear power plants. During this process, AE signals were measured using the AE testing system as the strain increased. Figure 5 shows the results of accumulated AE signals visualized according to the change in axial load. This figure helps clearly understand how AE signals accumulate under repeated loading. The Kaiser effect refers to the phenomenon where no AE signals are generated during loading if the applied load does not exceed the material’s previously experienced maximum load [34,35]. In contrast, the Felicity effect refers to the phenomenon where AE signals are generated even at loads lower than the previous maximum load, indicating instability related to crack propagation [36]. In ranges #1 and #2 of Figure 5, the Kaiser effect was observed at loads below 280 MPa, indicating that the cracks are growing stably (with minimal damage to the material). The occurrence of the Felicity effect after 280 MPa indicates that the cracks are growing unstably, which shows that damage to the material is progressing (#3, #4, #5, #6). In #5(3), AE signals are emitted during the unloading phase, which occurs because permanent deformation occurs in the weld cracks, leading to crack growth under compressive forces. Through these observations, the stability and instability of crack growth can be assessed, providing essential information for diagnosing the safety of structures.

**Figure 6:**
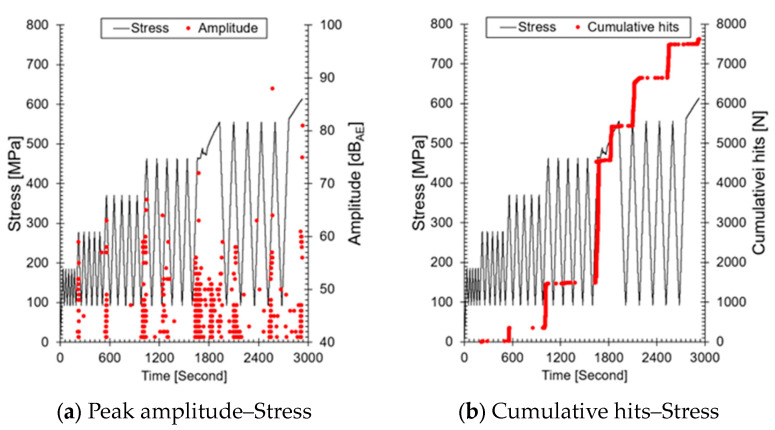
Representation of AE parameters under varying load conditions: (**a**) amplitude as a function of stress, reflecting the maximum size of AE signals; (**b**) cumulative AE hits as a function of stress, indicating the number of AE events during the test.

Figure 6 visually represents the AE parameters, amplitude, and AE hits, which are commonly used for crack growth monitoring, under varying load conditions. Amplitude refers to the maximum size of an AE signal, while AE hits represent the number of AE signals occurring within a specific time period. Amplitude is used to evaluate the size or nature of a defect through the signal’s magnitude, and when the highest amplitude is measured over a time series, it indicates the occurrence of a larger defect or crack compared to previous measurements.

AE hits represent the number of AE events occurring within a specific time period and indicate defect activity. An increase in cumulative AE hits (a rise in slope) can predict the occurrence of larger cracks. Therefore, as the step load increased, cumulative AE hits first increased in regions #3(1), #4(1), and #5(1), followed by the measurement of higher amplitude values compared to previous ones. This suggests that as the step load increases, the length and depth of the crack have increased compared to before. In the region after #3(1), where the Felicity effect appears, AE signals were emitted during loading, and after #5(1), signals were emitted even during the unloading phase. This result occurs when cracks form at the weld and continuous AE signals are emitted from the crack tip under repeated loading. The trends of amplitude and cumulative hits over time can be used to evaluate the progression of structural damage; this is applied in AE testing-based SHM. However, the structure we are targeting (ALMS installed in the primary system) uses only RMS, so this analysis method cannot be applied.

**Figure 7:**
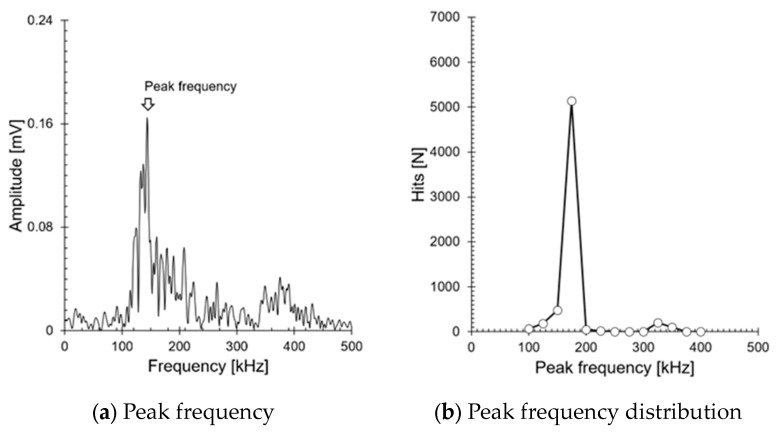
Peak frequency characteristics of AE signals: (**a**) peak frequency of AE signals; (**b**) frequency distribution (white circle) of AE signal peak frequencies measured during the test.

Figure 7 visually represents AE hits as a function of peak frequency, obtained by converting an 80 dB_AE_ signal using FFT (Fast Fourier Transform). This confirms that the cracks occurring at the welds have a single frequency range between 100 and 300 kHz. Numerous researchers have successfully evaluated crack growth stages using AE entropy, AF, RA, amplitude AE count, and energy, and have quantified the crack growth stages based on time-series changes in AE parameters [8,9,10,11,12,13,14,15,16]. Meanwhile, Graham and Alers reported that the amplitude of a 200 kHz AE signal decreases linearly with propagation distance during pressure vessel tests, but the rate of decrease is steeper near the signal source [6]. This is similar to the primary system welds we are targeting (inner diameter: 1066.8 mm). AE signals decrease non-linearly with propagation distance, and the reduction in AE parameters is inevitable depending on the distance between the sensor and the crack. Therefore, it is not possible to quantify the size of the crack using AE parameters alone. Specifically, in the primary system, a single AE sensor is installed near the weld to monitor abnormal conditions, so the attenuation of amplitude and AE hits is expected to be more significant. This indicates that in the ALMS system, which triggers an alarm when a certain threshold is exceeded, there is a possibility that the diagnosis could be amplified or minimized depending on the location of the crack. To address these issues, the *b*-value was adopted for evaluating weld cracks. The *b*-value is an indicator that quantitatively evaluates the crack progression by analyzing the distribution of AE events, representing the relationship between crack growth rate and the frequency of AE events. Through prior research [37], we confirmed that AE sources with a single frequency range are less affected by propagation distance. In contrast, when multiple frequency ranges are present, the *b*-value changes with propagation distance. This allows for a stable indication of abnormal conditions, as it displays the same values regardless of where a crack or leak occurs along the 3351.45 mm circumference of the weld line.

**Figure 8:**
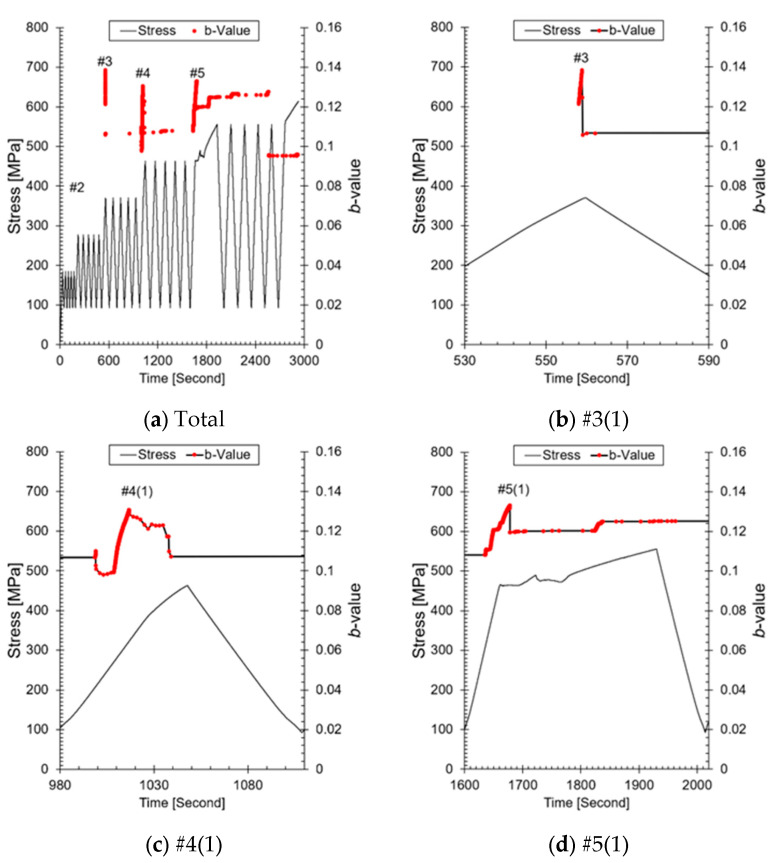
Amplitude-based *b*-value analysis: (**a**) total *b*-value calculated from AE signal amplitude; (**b**) *b*-value analysis at step #3(1); (**c**) *b*-value analysis at step #4(1); (**d**) *b*-value analysis at step #5(1), showing changes in crack progression.

In this study, traditional *b*-value techniques were used to perform AE analysis on metallic materials. This decision was made because it was determined that the correction phase could only proceed after the traditional *b*-value technique was validated in the AE signal analysis of metallic materials. Therefore, the traditional *b*-value technique was applied without correction to evaluate crack progression, and its applicability to metallic structures was examined. Figure 8 shows the results of the *b*-value calculated using amplitude and AE hits. The *b*-value is an indicator that quantitatively assesses the progression of cracks by analyzing the distribution of AE events and, unlike other parameters, reflects the stability and instability of crack growth. This is similar to evaluating the concentration and dispersion of AE signal intensity generated during the crack growth process compared to amplitude and AE hits [24,26,29,30]. An increase in the *b*-value indicates that the crack growth is slow or has stopped, suggesting that the energy related to the crack is being dispersed (micro-crack dominant). In contrast, a decrease in the *b*-value indicates that the crack is growing rapidly or that energy is being concentrated, which increases the risk of fracture (macro-crack dominant). Here, micro-cracking refers to the numerous small cracks that occur at the stress concentration area of the crack tip, while macro-cracks are relatively larger cracks that penetrate through the micro-cracks. Cracks grow by alternating between micro-cracks and macro-cracks. In Figure 8a, the *b*-value is displayed, and to observe the increase and decrease of the *b*-value, #3(1), #4(1), and #5(1) are shown again in Figure 8b–d. In all figures, the *b*-value increased when the previous maximum load was exceeded and then decreased, indicating that the crack transitioned from micro-cracks to macro-cracks (the size of the crack is larger than the previous one). As shown in the test results, the *b*-value changes accordingly with crack growth. Therefore, applying the *b*-value to the primary system welds allows for the evaluation of crack conditions, similar to amplitude and cumulative AE hits.

**Figure 9:**
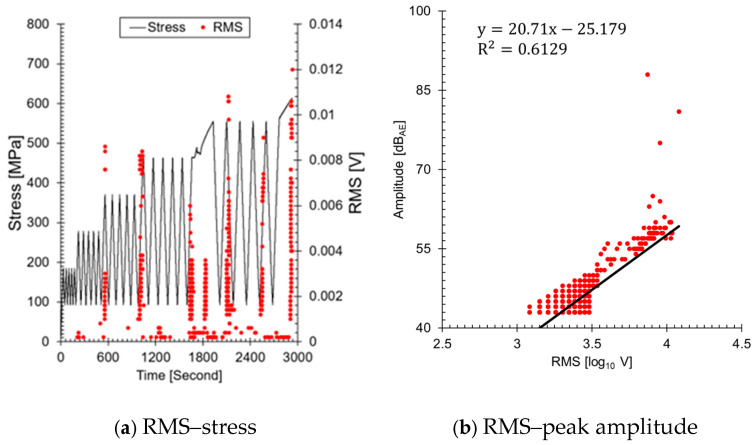
RMS characteristics of welded specimens: (**a**) RMS as a function of stress, reflecting the energy of AE signals; (**b**) RMS compared to peak amplitude, indicating the correlation between these AE parameters during crack progression.

**Figure 10:**
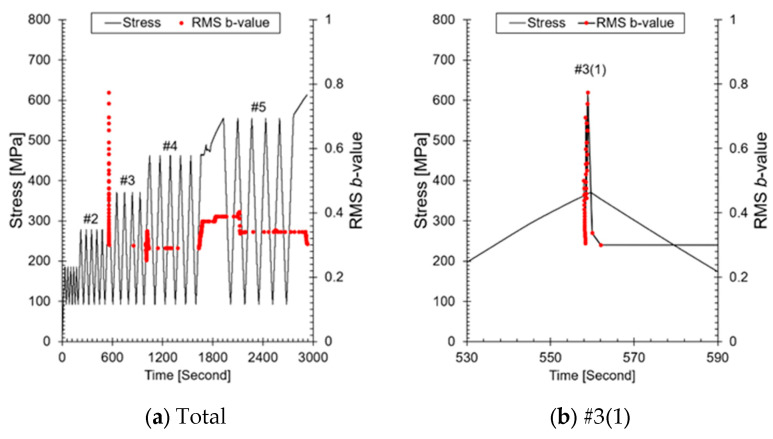

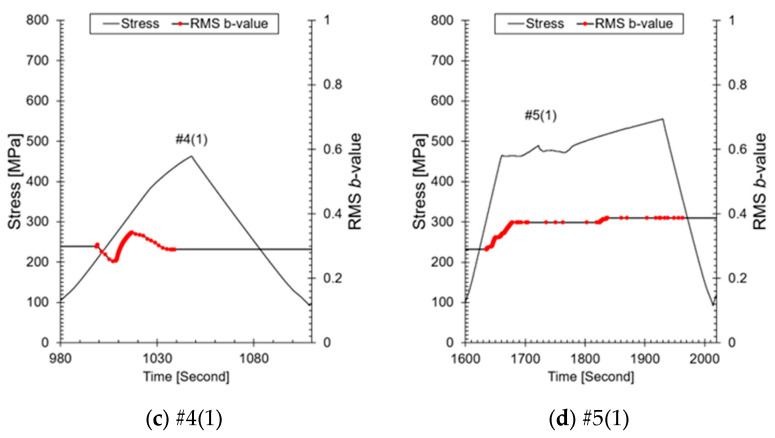
RMS-based *b*-value analysis: (**a**) total *b*-value calculated using RMS; (**b**) *b*-value analysis at step #3(1); (**c**) *b*-value analysis at step #4(1); (**d**) *b*-value analysis at step #5(1), demonstrating crack progression through RMS-based analysis.

In the ALMS used in the primary system, amplitude values that do not exceed the fault threshold are not recorded. Here, the threshold refers to the criterion for triggering the alarm and should be distinguished from the threshold used for measurements. Therefore, it is necessary to use other AE parameters for *b*-value analysis. Figure 9 shows the results of visualizing the RMS, an AE parameter stored to assess leaks in the ALMS. The reason both values increase similarly with changes in load is that both parameters reflect the energy of the AE signals. RMS represents the total energy of the AE signal, while amplitude reflects the instantaneous maximum value of the signal. As the load increases, the energy emitted from defects within the material also increases, leading to a similar increasing trend in both parameters (Figure 9b). However, it is important to note that the data are subject to limitations due to the use of an amplitude-based threshold during the measurement phase. This threshold ensures that only signals exceeding a certain amplitude are recorded, which could result in the underrepresentation of lower energy AE events that might still be significant for *b*-value analysis. Consequently, this may have influenced the correlation (0.6129) between amplitude and RMS values, as well as the overall assessment of crack progression. Therefore, during the calculation of the *b*-value, amplitude was replaced with RMS; the results are presented in Figure 10a. The *b*-value calculated based on RMS shows a similar trend to that calculated based on amplitude (see Figure 8). To observe the increase and decrease of the RMS-based *b*-value, #3(1), #4(1), and #5(1) were highlighted again in Figure 10b–d. The RMS-based *b*-value increased when micro-cracks grew at the crack tip and decreased when macro-cracks occurred. These results are consistent with the analysis based on amplitude and cumulative AE hits. If the RMS-based *b*-value is applied to the drain valve event mentioned in the introduction, it is expected to serve as a fundamental datum for assessing the growth and reduction of micro-cracks before leakage. The results of this study demonstrate the potential to monitor and assess the crack status of nuclear power plant piping in real time using AE data. In particular, it suggests a method to enhance the safety of nuclear power plants and quantitatively understand crack progression by adding a simple AE analysis to existing methods. However, there is a limitation in that the relationship between crack growth, RMS, and the *b*-value has not been experimentally proven. This aspect will be pursued in future research.

## 4. Conclusions

This study presents a method to add a crack analysis algorithm to the ALMS system to detect and evaluate the crack growth process in the primary system piping of nuclear power plants. To achieve this, a fracture test was conducted by applying stepwise loading to welded specimens that simulate the cold leg section, and AE signals were measured using an AE testing system in relation to the increase in strain.

First, the experimental results indicated that the stability and instability of cracks could be assessed through the Kaiser effect and the Felicity effect in detecting crack growth using AE signals. The Kaiser effect was observed when the crack was growing stably, while the Felicity effect indicated unstable crack growth, confirming that damage to the material was progressing.

Second, by utilizing the parameters of amplitude and AE hits, it was possible to quantitatively assess the size and activity of the cracks. The increase in amplitude as the experiment progressed indicated that the crack size was increasing and that more energy was being released. Additionally, AE hits reflect the rapid progression of activated cracks and the cumulative AE hits allowed for the assessment of the severity of the defects.

Third, the *b*-value was introduced for weld crack monitoring. The *b*-value was effective in assessing the crack growth rate and energy dispersion through the distribution of AE events. Specifically, an increase in the *b*-value indicates a dominant state of micro-cracking, while a decrease signifies a concentrated growth of macro-cracks. This analysis provides a method to accurately understand the progression of cracks while minimizing assessment errors caused by AE signal attenuation.

Finally, the results of calculating the *b*-value using both the RMS and amplitude parameters confirm that the RMS-based *b*-value minimizes the effects of AE signal attenuation and allows for a more stable assessment of crack progression. This demonstrates that the RMS, which reflects signal energy, is effective for real-time monitoring of the crack growth state.

The results of this study suggest the possibility of real-time crack monitoring using AE data in piping systems of critical structures such as nuclear power plants. In particular, by adding a simple AE analysis method to the ALMS system, a practical approach has been derived that enhances the safety of the structure and allows for quantitative assessment of crack progression. Further studies will combine RMS-based *b*-value analysis with cumulative AE energy analysis to more effectively track crack severity and damage progression. This approach aims to develop a real-time monitoring system capable of predicting critical defect occurrence. The results are expected to contribute to further refinement of AE parameters and algorithms, leading to advancements in safety monitoring systems across various industrial settings.

## Figures and Tables

**Table 1:** Test conditions for AE testing.

	Threshold	Amplified	Analog Filter		Sampling Condition		
Type	dB_AE_	dB_AE_	Lower	Upper	Rate	PDT	HDT
Fixed	40	40	1 kHz	1 MHz	10 MHz	50 μs	1.5k μs

## Data Availability

The original contributions presented in the study are included in the article, further inquiries can be directed to the corresponding author.
